# Anticancer activity of 23,24-dihydrocucurbitacin B against the HeLa human cervical cell line is due to apoptosis and G2/M cell cycle arrest

**DOI:** 10.3892/etm.2021.10033

**Published:** 2021-04-12

**Authors:** Jun-Xiao Zhang, Wei-Tan Hong, Chun-Yan Hu, Wei-Qiang Wang, Guang-Hua Chu, Li-Hui Wei, Liu Chen

Exp Ther Med 15:2575-2582, 2018; DOI: 10.3892/etm.2018.5710

Following the publication of the above paper, a concerned reader drew to the Editor’s attention that several figures contained data that bore striking similarities to data published in other papers; notably, the flow cytometric data shown in Fig. 4A appeared to be identical with data published in the original (and now archived) version of another paper published around the same time, although by different authors from different research institutions [Lin J, Zhao Y, Shi Z and Bai Y: Antitumor effects of helenalin in doxorubicin-resistant leukemia. JBUON 24: 2068-2074, 2019].

After having conducted an independent investigation in the Editorial Office, the Editor of *Experimental and Therapeutic Medicine* has determined that this article should be retracted from the Journal on account of a lack of confidence concerning the originality and the authenticity of the data. The authors were asked for an explanation to account for these concerns, but the Editorial Office never received any reply. The Editor regrets any inconvenience that has been caused to the readership of the Journal.

